# Pigeons integrate visual motion signals differently than humans

**DOI:** 10.1038/s41598-019-49839-x

**Published:** 2019-09-16

**Authors:** Yuya Hataji, Hika Kuroshima, Kazuo Fujita

**Affiliations:** Kyoto University, Department of Psychology, Graduate School of Letters, Kyoto, Japan

**Keywords:** Sensory processing, Psychology

## Abstract

Perceiving motion is a fundamental ability for animals. Primates integrate local 1D motion across orientation and space to compute a rigid 2D motion. It is unknown whether the rule of 2D motion integration is universal within the vertebrate clade; comparative studies of animals with different ecological backgrounds from primates may help answer that question. Here we investigated 2D motion integration in pigeons, using hierarchically structured motion stimuli, namely a barber-pole illusion and plaid motion. The pigeons were trained to report the direction of motion of random dots. When a barber-pole or plaid stimulus was presented, they reported the direction perpendicular to the grating orientation for barber-pole and the vector average of two component gratings for plaid motion. These results demonstrate that pigeons perceive different directions than humans from the same motion stimuli, and suggest that the 2D integrating rules in the primate brain has been elaborated through phylogenetic or ecological factors specific to the clade.

## Introduction

Perception of motion is fundamental for animals as it provides information about the presence of prey, predators, mating rivals, and partners. Visual motion perception is realized by hierarchical stages of motion processing^1^. The most basic stage of motion processing detects a spatiotemporal change of visual cues (primarily luminance) within local receptive fields (1D motion). Because of its computational characteristics, 1D motion is ambiguous in itself. There is always a family of physical movements in two dimensional space that produce the same local 1D motion, and these possible movements lie on the constraint lines in two dimensional vector space along the orientation of 1D motion detector^2^. The second stage of motion processing integrates these 1D features across orientation and spatial receptive field to solve this motion ambiguity and compute a single rigid motion in the 2D space (2D motion).

**Figure 1:**
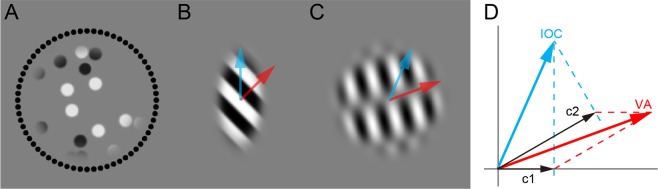
Illustrations of the stimuli used in this study. (**A**) A random dot motion stimulus used for the discrimination task of motion direction, with surrounding black response dots. (**B**) A barber-pole stimulus used in Experiment 1 (window ratio is 2:1). The oblique grating (red arrow) is perceived to move along the ellipse window (blue arrow) by integrating rigid 2D motions included in bar ends. (**C,D**) A plaid stimulus used in Experiment 2 (relative motion direction is 30°). Red and blue arrows indicate two possible solutions of motion integration: the intersection of constraint lines (IOC) and the vector average (VA).

The rule of 1D motion integration across orientation has been explored using plaid motion stimuli, in which two drifting gratings in different orientations are superimposed^2^ (Fig. 1C). Different computational solutions have been proposed to reconstruct the 2D motion from 1D motion components of plaid^2^: intersection of constraints (IOC) or vector average (VA) (Fig. 1D). The IOC solution is the unique vector consistent with both 1D components and defined as the intersection of constraint lines of both 1D component motions. The VA solution is the average of normal vectors of both components. Although the VA solution is dominant for humans in situations such as peripheral viewing or brief presentation of stimuli^3^, humans typically perceive plaid motion in the IOC direction^2^. This indicates that humans have multiple solutions to integrate local 1D motion and, when foveally viewing attentively, humans depend on the IOC rule.

The barber-pole illusion is another stimulus that has been used for investigating the rule of 1D motion integration across visual space^4^ (Fig. 1B). When seeing a diagonally drifting grating through a vertical ellipse window, we perceive it as moving vertically. Although several theories have been proposed for the barber-pole illusion^5,6^, one powerful hypothesis, the end-stop theory, assumes that rigid 2D motions in peripheral grating terminators are spatially combined with central ambiguous 1D motions to produce a unified 2D motion vector^7,8^.

To better understand the evolutionary history of visual motion system it is important to investigate whether these rules of integration from 1D to 2D are shared across primate and non-primate species. Here we focus on avian species, because most of them have a well-developed but totally different visual system than that in the primate brain. Previous studies (mostly on pigeons) have demonstrated that avian species are sensitive to visual motion: they can distinguish various motion patterns such as the speed and direction of stripes^9,10^, global dot motion pattern, object motion, and biological motion^11–17^. Physiological studies have found that visual motion is processed across the different visual processing stages in the pigeon brain (e.g. optic tectum^18^, nucleus rotundus^19^, entopallium^20^). Two physiological studies have investigated 2D motion integration in avian species. Neurons in owl’s visual Wulst respond to 1D components of plaid as in mammalian primary visual cortex^21^. Two nuclei in the pigeon’s accessory optic system that processes optic-flow caused by self-motion are selective for integrated 2D motion of plaid^22^.

In the present study we investigated how pigeons (*Columba livia*) integrate local 1D motion to perceive 2D motion in behavioral experiments using two kinds of hierarchical motion stimuli: a barber-pole illusion^4^ and plaid motion^2^ (Fig. 1). First, we trained pigeons to report the direction of moving random dots by pecking clock-face response dots surrounding the motion stimulus (Fig. 1A). After confirming that the performance was above chance, we presented barber-pole and plaid stimuli as probes in two different experiments to test whether responses to these probe stimuli were consistent with 2D-defined directions (Fig. 1B,C).

## Results

### Pigeons do not see a barber-pole illusion

We tested a barber-pole illusion in pigeons using two aspect ratios of stimulus window (1:1 and 1:2). The grating and window orientations were independently manipulated. If the pigeons perceive a barber-pole illusion via spatial integration of local motion signals, their response directions should be biased toward the direction of the major axis in the ellipse window condition, particularly when the grating orientation is diagonal to the window orientation, whereas in the circular window condition it should be consistent with the grating direction.

In training on discrimination of moving direction with random dots, four male pigeons performed at 56.4 to 87.2% correct in the final three sessions, far above chance level (25%). This baseline performance remained excellent when probe trials were inserted in test sessions (56.6–81.4%). Accuracy did not change drastically for different target directions and secondary stimulus factors, such as dot size and speed, ensuring that the pigeons performed based on motion direction (see Supplementary Fig. S1).

**Figure 2:**
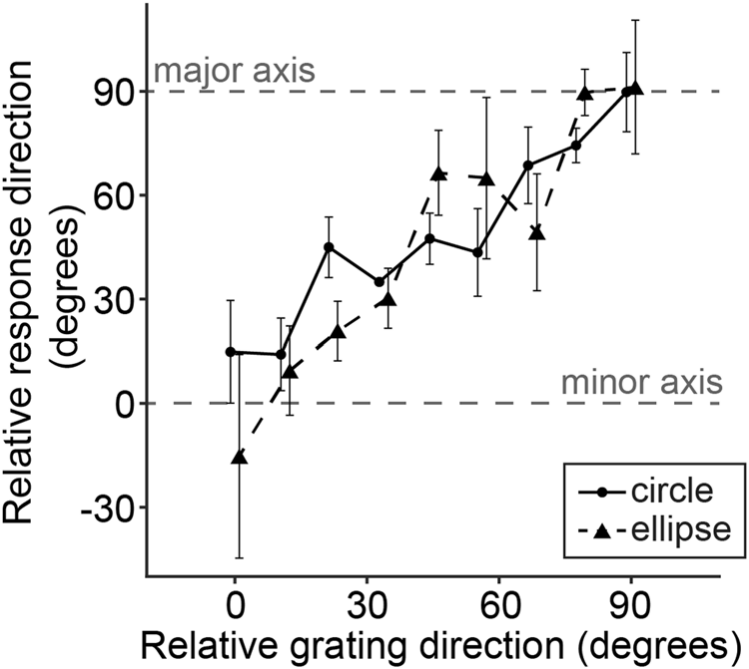
Circular means of pigeons’ response directions toward barber-pole stimulus relative to the window orientation as a function of relative grating direction. The solid and dotted black lines indicate circular and ellipse window conditions, respectively. The upper and lower gray dotted lines indicate the major and minor axis of ellipse window, respectively. Error bars indicate SEM.

In the probe task with the barber-pole stimulus, pigeons responded to the grating directions not linked to the major axis direction in the ellipse window condition (Fig. 2). There was a significant main effect of grating direction (Harrison-Kanji test, *F*(8, 54) = 5.96, *p* < 0.001, *η*^2^ = 0.42). The window ratio had no effect on response directions (*F*(1, 54) = 1.85, *p* = 0.18, *η*^2^ = 0.02 for the main effect of window ratio; *F*(8, 54) = 0.58, *p* = 0.79, *η*^2^ = 0.04 for the interaction between window ratio and grating direction). This result indicates that pigeons do not see a barber-pole illusion, suggesting that motion perception in pigeons is insensitive to rigid 2D motions in peripheral end-stops. This suggestion is also supported by the fact that the pigeons’ response directions were not biased toward the major axis as the spatial frequency and the number of grating end-stops increased (see Supplementary Fig. S2).

### Pigeons see a plaid motion in the vector average direction

In contrast to two previous studies of birds using Type I plaids, composed of two gratings of the same speed^21,22^, we here used Type II plaids, in which the IOC and VA directions are segregated^2^ (Fig. 1C,D). The temporal frequency of one component was twice that of the other component, and the differences of component orientations were set from 15 to 90°. If the pigeons responded to this plaid stimulus based on the IOC solution, their responses should be directed outside of faster component, particularly when the difference of component directions was small. If the pigeons responded based on the VA solution, their responses should be directed between the components.

Three pigeons from the previous experiment and two naive pigeons were trained on the same motion discrimination as in the previous experiment. The pigeons performed at 62.1 to 74.7% correct in the final three training sessions before advancing to the test sessions. Performances in training trials were not weakened by inserting probe trials in test sessions, and performances of naive individuals were comparable to those of older individuals (naive: 75.3, 65.9%; older: 70.7, 66.5, 66.4%). Accuracy was above chance level across target direction and secondary stimulus factors (see Supplementary Fig. S3).

**Figure 3:**
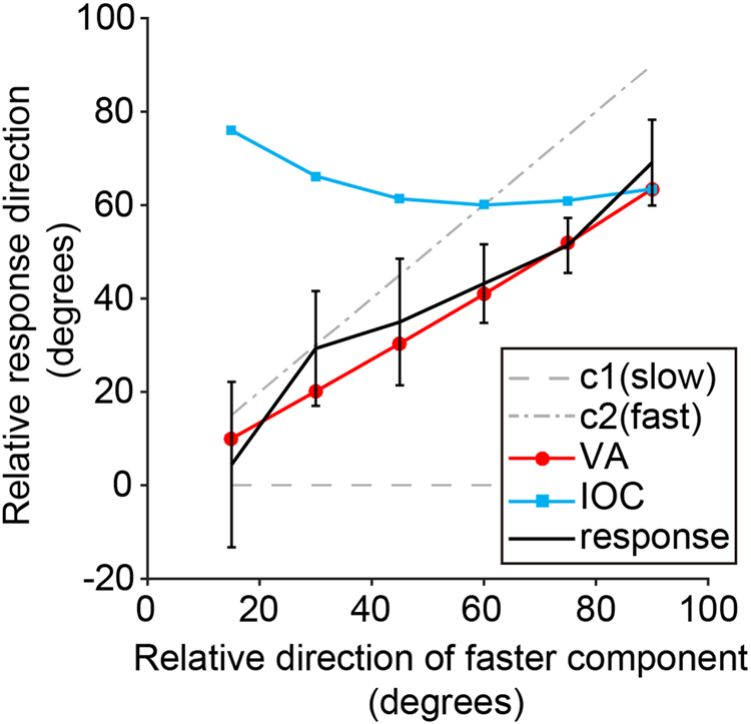
The pigeons’ responses were consistent with VA direction of plaid stimulus. A black line with error bars indicates circular means and SE of response direction relative to the component 1 direction calculated from circular means of individual data. Red and blue lines represent directions of vector average (VA) and intersection of constraint (IOC) for each faster component direction.

Figure 3 shows response directions toward the plaid stimuli as a function of the faster component direction relative to the slower component direction. The birds responded following the VA directions, not the IOC directions. We fitted a linear mixed model to estimate whether VA or IOC predicted the pigeon’s response direction (Table 1). We found a significant effect of VA (*p* < 0.0001). A likelihood ratio test revealed that adding the effect of IOC did not better model the pigeon’s response (Table 2). These results indicate that pigeons responded not to the IOC but to the VA.

**Table 1:** Model output from linear mixed model predicting the pigeon’s response direction to plaid motion.

factor	estimate	s.e.	DF1	DF2	*F* stat	*p*-value
(Intercept)	0.101	0.029				
IOC	−0.034	0.051	1	2847	0.46	0.50
VS	0.593	0.081	1	2847	53.18	3.92 × 10^−13^

VA and IOC directions were used as fixed factors and subject ID was used as random intercept factors.

**Table 2:** Result of likelihood ratio test.

Model	DF	AIC	BIC	LR stat	deltaDF	*p*-value
Response ~ 1 + VS + (1|Subject)	4	10580	10604			
Response ~ 1 + IOC + VS + (1|Subject)	5	10582	10611	0.455	1	0.500

A LMM model with the fixed effect of VA was compared to a model with the effects of VA and IOC to investigate whether adding IOC value better modeled the pigeon’s response.

To eliminate the possibility that the pigeons just responded to either component motion and this resulted in the mean response to VA direction, we analyzed whether pecking variance increased as the difference of component orientations increased. If the pigeons simply responded to the components, the pecking variance would increase as the difference of component orientations increased. Contrary to this hypothesis, pecking variance decreased as the difference of component orientations increased, indicating that the pigeons responded to an integrated 2D motion (see Supplementary Fig. S2).

## Discussion

This study used barber-pole and plaid stimuli to investigate the integrating processes from 1D to 2D visual motion in pigeons. Two behavioral experiments demonstrated that pigeons do not see the same motion directions as primates from these hierarchically structured stimuli. Pigeons do not see a barber-pole illusion in a diagonally oriented grating through an ellipse window. Pigeons perceive motion consistent with the VA solution from a plaid motion, whereas primates typically perceive motion consistent with the IOC solution.

These results have implications for different characteristics of 2D integration of motion in pigeons compared to primates. First, from the result of the barber-pole experiment, the spatial limit of 2D motion integration is smaller in pigeons than in primates. This is consistent with the previous finding that pigeons tend to attend local visual features when discriminating visual stimuli^23^ and only a part of information within a visual receptive field is conveyed to higher visual processing stages^24^. Second, from the result of plaid experiment, the pigeons’ responses toward VA directions could be due to the lack of a higher-order motion system, which is necessary for the IOC solution in primates^25^. This is unlikely, however, because higher-order motion systems are present in fish^26^ and flies^27^, and are considered to be a shared system across sighted animals. Rather, sensitivity or integration weight of higher-order motion is likely to differ between pigeons and primates.

We consider some potential confounds that might have affected the data: viewing duration, viewing distance and peripheral viewing. It could be argued that the viewing duration was very short, because previous studies indicate that the perceived direction of moving bar is orthogonal the bar orientation at motion onset^28^, and that plaid motion is perceived to move in VA direction for brief presentations^25^. In our study, motion stimulus appeared 2 sec ahead of the response dots, and during this delay the pigeons looked at and pecked the stimulus, although not required in our task. Thus, viewing time before the pigeons made their decisions was sufficient. Another potential issue is viewing distance. This is always a problem in animal studies because viewing distance with a touch monitor is much smaller than in typical psychophysical experiments with humans^29^. Our barber-pole stimulus may have been too large in visual angle to attend to peripheral 2D motion. The temporal and spatial frequency of the plaid stimulus from the pigeon’s view may not be appropriate for the higher-order motion system to work^30^. The third possible artifact is related to the second one: human experiments indicate that higher-order motion does not work with peripheral viewing^3^. In the current experiment the pigeons looked at the motion stimulus from close distance in their frontal visual field with temporal foveas. In contrast, pigeons typically look at distant objects in their lateral visual field with central foveas^31^. The density of retinal ganglion cells is higher in a pigeon’s central fovea compared to a temporal fovea^32^, and velocity thresholds are smaller with lateral than frontal visual fields^33^. One solution to the second and third problems might be to separate the computer display from the testing chamber to control visual angle and induce the use of lateral visual field. This would be challenging, however, because the response toward the visual target is limited and discrimination learning is difficult in such a setup^29^.

If the present findings reflect differences in integration rules of 2D motion between pigeons and primates, why are they different? One possible reason is that the two species use different dominant visual pathways^34^. There are several visual pathways from retina to telencephalon in vertebrates. Primates largely depend on the thalamofugal pathway, which relays information from retina to the caudal part of telencephalon through the dorsal lateral geniculate nucleus. In contrast, the primary visual pathway for pigeons is the tectofugal pathway, which relays information to the optic tectum in the midbrain^34^. Blindsighted people with damage in the thalamofugal pathway still discriminate visual features based on the tectofugal pathway, and a version of the line motion illusion^35^ is perceived to move in a different direction by humans with blindsight^36^. This suggests that visual motion is processed in the different ways between thalamofugal and tectofugal pathways. Crowder and Wylie found that neurons in the third visual pathway of the pigeon’s brain related to the processing of optical flow respond to integrated directions of type I plaid^22^. They proposed that these neurons are sensitive to the integrated motion of plaid because the retinal inputs are orientation-insensitive, as compared to visual Wulst in the thalamofugal pathway^37–40^, which is analogous to primary visual cortex of mammals. Neurons in optic tectum of the pigeon brain also lack tuning for line orientation^41,42^. Therefore, although both thalamofugal and tectofugal pathways have direction-selective neurons, shapes of receptive field to compute local motion could differ between these pathways. To reveal a proximate factor in the species difference of motion integration reported here, further work should focus on the difference in motion processing between thalamofugal and tectofugal pathways.

The species difference in integration rules of 2D motion may reflect functional differences related to visual processing between the species. Although visual motion information is utilized for a diversity of internal processes, precise information about motion is required particularly for tracking a freely moving object. Primates can smoothly track an object using motion information with their eyes^43^, and hands^44^, whereas pigeons close their eyes during pecking^45^. Therefore they are thought to lack these smooth motor responses. For pigeons, feeding on stationary seeds on the ground^46^, smooth visuo-motor controls using precise information of motion may be less important, at least in foraging contexts, compared to carnivorous and omnivorous species that hunt rapidly moving preys.

For testing hypotheses about species difference in 2D integration of motion similar experiments should be done with other species. In particular, owls are of interest because they are carnivorous and have similar architectures in their visual system to primates, despite their phylogenetic distance^21,39,40^. It is also important to test non-primate mammals relying less on the thalamofugal pathway (e.g., rodents^47^) to reveal how 2D integration of motion occurs in various species of mammals, thus clarifying phylogenetic and ecological factors that affected 2D motion perception.

## Conclusions

This study demonstrated that pigeons perceive different motion directions than primates from barber-pole and plaid stimuli. Whereas primates perceive motion along the ellipse window of a barber-pole stimulus and in the IOC direction of a plaid stimulus, pigeons responded to the grating orientation of a barber-pole and to the VA direction of a plaid. This suggests that the rule of 2D integration of motion utilized by primates is not shared with all vertebrates and evolved though specific phylogenetic or ecological factors.

## Methods

### Experiment 1 (barber-pole illusion)

#### Subjects

Four male pigeons participated. They had participated in several unrelated studies, with two having participated in an earlier motion discrimination study^48^, in which they discriminated the rotating direction of single dot within a white circle. They were individually housed and maintained at 80–90% of their free-feeding weights. Water and grit were freely available in the home cage. The experiment adhered to the ethical guidelines of Kyoto University, and was approved by the Animal Experiments Committee of the Graduate School of Letters, Kyoto University (No. 17–46).

#### Apparatus

Each bird was tested in an operant chamber (W/D/H was 41/31/40 cm) installed with a 15-in. LCD monitor (EIZO, FlexScan L357) with 1024 by 768 pixels resolution running at 60 Hz refresh rate and a touch sensitive frame (MINATO HOLDINGS, ARTS-015N-02B). A grain hopper delivered food rewards through an opening on left-side wall located about 26 cm from the center of display monitor. Fluorescent room lights illuminated the apparatuses and the apparatus had no attached houselight. The experiments were controlled by a personal computer (Mouse Computer, LM-i500SC, or ThirdWave Corporation, Diginnos Series) running MATLAB with the Psychtoolbox extensions^49^.

#### Stimuli

For training stimuli, white and black dots moved in the same direction within a circular window (89.1 mm diameter, Fig. 1A). The number, size, contrast and speed of dots were randomly determined in every trial to prevent learning based on potential local information (number: 20–40, 1 increments; size: 4.5–10.4 mm, 0.3 increments; contrast: 0.6–1.0, 0.01 increments; speed: 71.3–142.6 mm/s, 17.8 increments). The motion direction had 32 conditions (11.25° increments from upper direction).

For probe stimuli, a drifting grating was presented within a circular window (89.1 mm diameter) or an elliptical aperture window (aspect ratio was 2:1, 89.1 mm diameter for the major axis, Fig. 1B). The grating was made by modulating the contrast of sinusoidal grating by a cumulative Gaussian function (μ = 0.5, σ = 0.12) to enhance edge appearance. The speed of the grating was 71.3 mm/s. The spatial frequency and contrast of the grating was randomly determined in every trial (spatial frequency: 8.9–20.8 mm/cycle; contrast: 0.6–1.0, uniformly distributed). The motion direction of grating had 32 conditions (11.25° increments from upper direction). The orientation of ellipse window had 8 conditions (22.5° increments from vertical orientation).

#### Procedure

The pigeons were trained to report perceived direction of a motion stimulus. A trial started after the pigeon pecked a self-start key (14.9 by 14.9 mm white square) on a gray back ground. A motion stimulus appeared at the center of the display, and 2 s later, 64 black response dots appeared around the motion stimulus (Fig. 1A). The pigeons got rewarded if they pecked the response dots three times in the same direction as the motion direction (range was ±45°, hence chance level was 25%). Access to the food was granted with a probability of 50–60%. Regardless of the delivery of the food, the light above the food hopper came on when the subject’s response was correct, serving as a secondary reinforcement. Pecking three times to the other dots resulted in 5–7 s timeout and a correction trial was inserted before the next trial. In a correction trial, the same stimulus appeared and pecks to the wrong dots were not counted. This treatment aimed to prevent response biases. The duration of timeout, access to the food, and the probability of secondary reinforcement varied according to the weight and motivation of each subject (timeout: 5–7 s; food access: 2.8–5 s; secondary reinforcement: 50–60%).

Daily training session consisted of 224 training trials with 32 conditions about dot motion directions. Each bird completed at least 51 training sessions before advancing to test sessions. After confirming that performance was above chance (25%), the subjects advanced to test sessions. Daily test sessions consisted of 192 training trials and 32 probe trials. The training stimuli had 32 dot motion directions. The probe stimuli had 32 conditions for grating direction, 8 conditions for orientation of aperture window, and 2 conditions for window aspect ratio (circle or ellipse). Each subject responded to 512 combinations of probe stimuli randomly distributed with respect to all three parameters in 16 sessions (thus, parameters were not counterbalanced in a session). In probe trials, the angle of correct area was randomly determined irrespective of grating direction, and width of the correct area was determined so that its ratio corresponded to the percentage correct in the training trials of the last session. For example, when the percentage correct in the training trials of the last session was 60%, the width of correct area in the probe trials of the present session was set to ±108° around the randomly determined correct direction. This treatment was to equate reinforcement rates between training and probe trials. Between test sessions, at least one training session was conducted to prevent degradation of performance due to the addition of probe trials.

#### Analysis

We converted target and response directions so that 0° represented the minor axis of ellipse window (or pseudo major axis of circle window) and directions were folded into the first quadrant, which resulted in 9 target directions (e.g. 45, 135, 225 and 315° were all converted to 45°). For probe trial data, circular means of the pigeon’s relative response directions were calculated for each subject, window ratio and target relative direction. A two-way Harrison-Kanji test, which is analysis of variance for circular data, was applied to the individual mean data to analyze the effects of relative target direction, window ratio and their interaction on response directions^50^.

### Experiment 2 (plaid motion)

#### Subjects and apparatus

Five male pigeons participated, three of which had participated in Experiment 1. Two new pigeons had been shaped to peck to the touch screen but were naive to discrimination tasks. Five operant chambers were used as apparatus. The other details of subjects and apparatus were the same as Experiment 1.

#### Stimuli

The training dot stimulus was the same as that in Experiment 1 except that the ranges of stimulus parameters changed (size: 5.94–11.88 mm, uniformly distributed; speed: 17.8–71.3 mm/s, uniformly distributed). The dot contrast was fixed at 1.0. For probe stimulus, two drifting gratings of different directions were superimposed. The spatial frequency and contrast of both component gratings was 11.9 mm/cycle and 1.0. The speed was set to 17.8 mm/s for one component and 35.6 mm/s for the other component. These unequivalent speeds for two components separated two possible integrated directions: vector average (VA) and intersection of constraints (IOC). VA is the vector average of the two components. Due to the aperture problem, each component grating has an infinite combination of direction and speed that lies on a constraint line in the vector space. IOC is the direction that corresponds to the intersection of constraint lines of two components. The motion direction of the slower component had 16 conditions (22.5° increments from upper direction). The motion direction of the faster component relative to the slower one had 12 conditions (±15–90°, 15°increments).

#### Procedure

In a training session with 192 trials, random dots motion with 16 motion direction conditions were presented. In a test session with 192 trials, 16 trials were probes. The probe stimuli had 16 conditions for slower component direction, 12 conditions for the faster component direction relative to slower one (±15–90°, at 15° intervals). Each subject responded to 192 combinations of probe stimuli in 12 test sessions.

#### Analysis

For the probe trial data we rotated target and response directions so that 0° represented the direction of slower component and the response direction ranged from −180 to 180°. To test whether IOC, VA or both contributed to the response direction, a linear mixed model was fitted to response directions, with IOC and VA directions as a fixed factor and subject as a random intercept factor using the ‘fitglme’ function embedded in MATLAB.

## Supplementary information

Supplementary information

## Data Availability

The data reported in this paper have been deposited in Kyoto University Research Information Repository (http://hdl.handle.net/2433/242241).
