# Supplementary material for: Pigeons integrate visual motion signals differently than humans
Source: Sci Rep. 2019 Sep 16;9:13411. doi: 10.1038/s41598-019-49839-x (PMC6746846; doi:10.1038/s41598-019-49839-x)
Supplement: Supplementary file 1 — Supplementary information [file 41598_2019_49839_MOESM1_ESM.pdf]

Supplementary Information

Pigeons integrate visual motion signals differently than humans

Yuya Hataji, Hika Kuroshima, and Kazuo Fujita

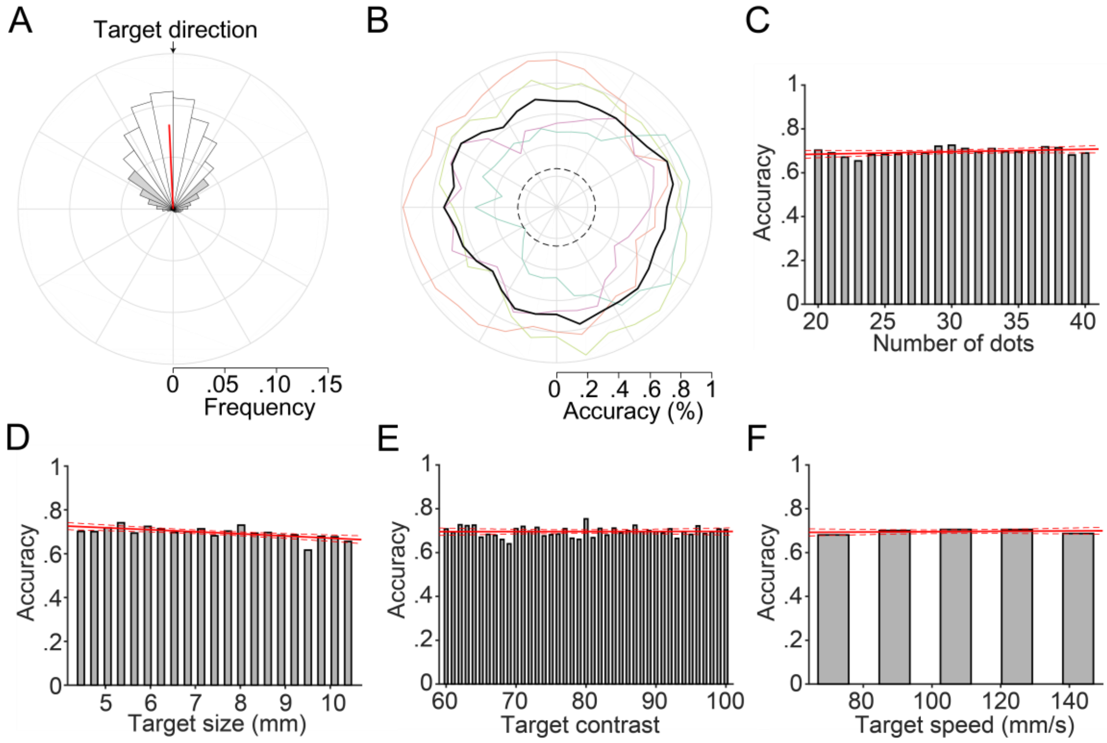

Supplementary Figure S1. The pigeons' performance of baseline trials in Experiment 1.

(A) Distribution of pecking direction relative to the target direction. White and gray bars were counted as correct and wrong responses. A red line indicate the circular mean of pecking direction. (B) Accuracy for each target direction. Colored thin lines and black thick line indicate individual and summed performance. A dotted line indicates chance level (25%). (C-F) Effects of secondary stimulus factors on the pigeon's performance. Red lines indicates regression lines fitted with GLM.

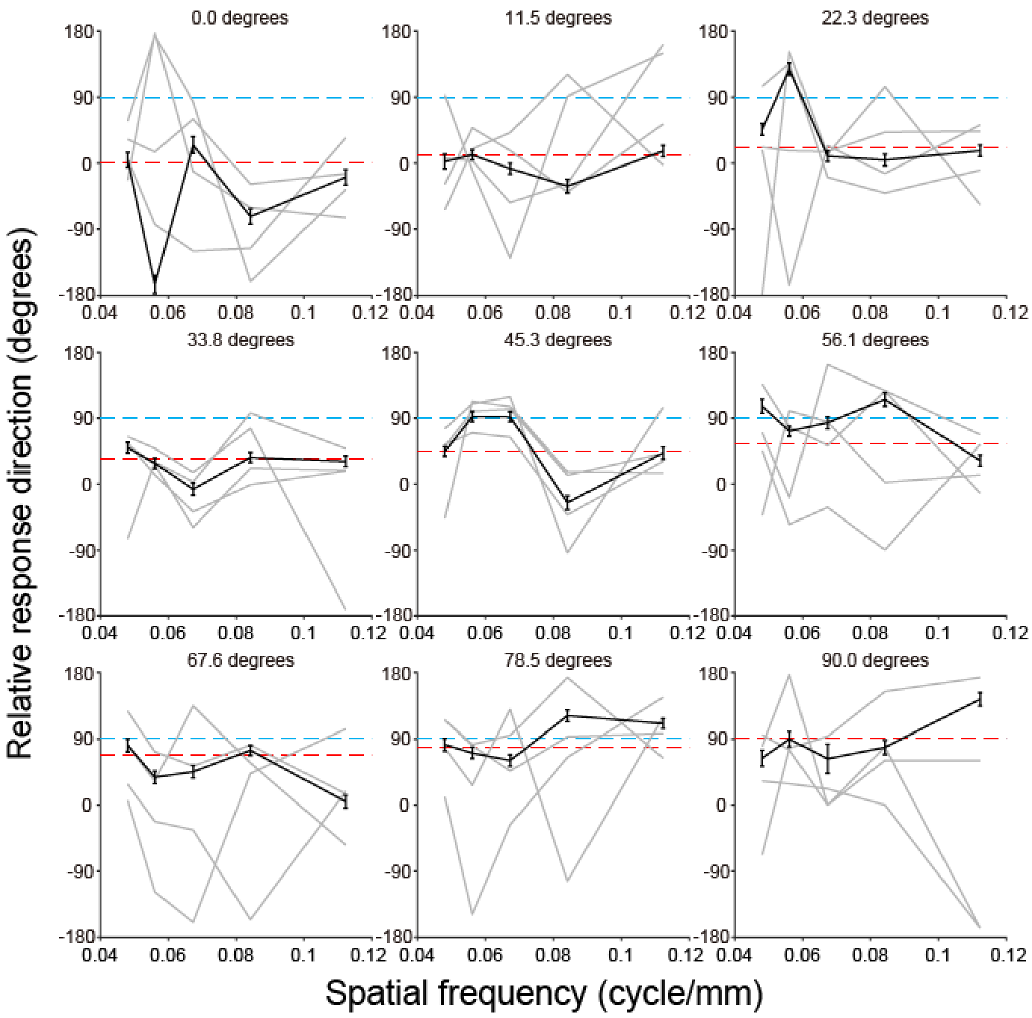

Supplementary Figure S2. The pigeons' responses were not biased as the number of bar end increased in the ellipse window condition. Response directions relative to the minor axis of ellipse window were plotted as a function of spatial frequency of grating. Each panel represents a grating direction relative to the minor axis of ellipse. Black lines represent circular means with SEM from all subject data. Gray lines represent circular means for each subject. Red lines represent relative grating directions. Blue lines represent the major axis of ellipse, which is the direction of barber-pole illusion.

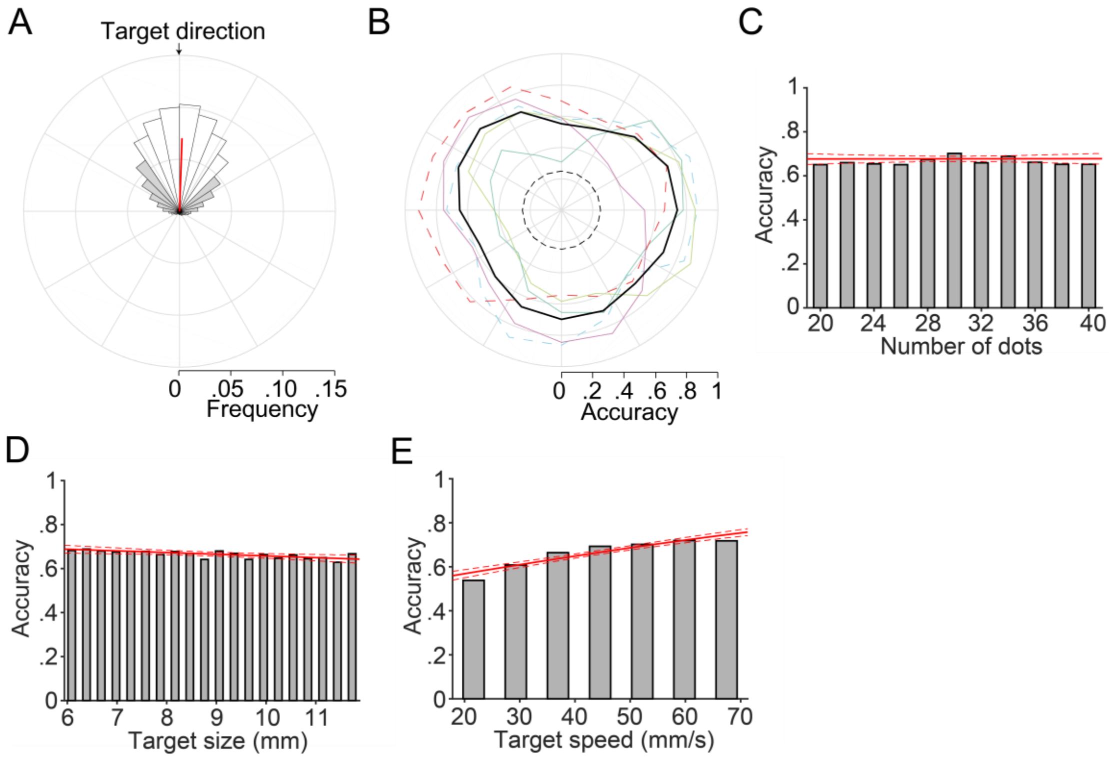

Supplementary Figure S3. The pigeons' performance of baseline trials in Experiment 2.

(A) Distribution of pecking direction relative to the target direction. White and gray bars were counted as correct and wrong responses. A red line indicate the circular mean of pecking direction. (B) Accuracy for each target direction. Colored thin lines and black thick line indicate individual and summed performance. A dotted black line indicates chance level (25%). (C-E) Effects of secondary stimulus factors on the pigeon's performance. Red lines indicates regression lines fitted with GLM.

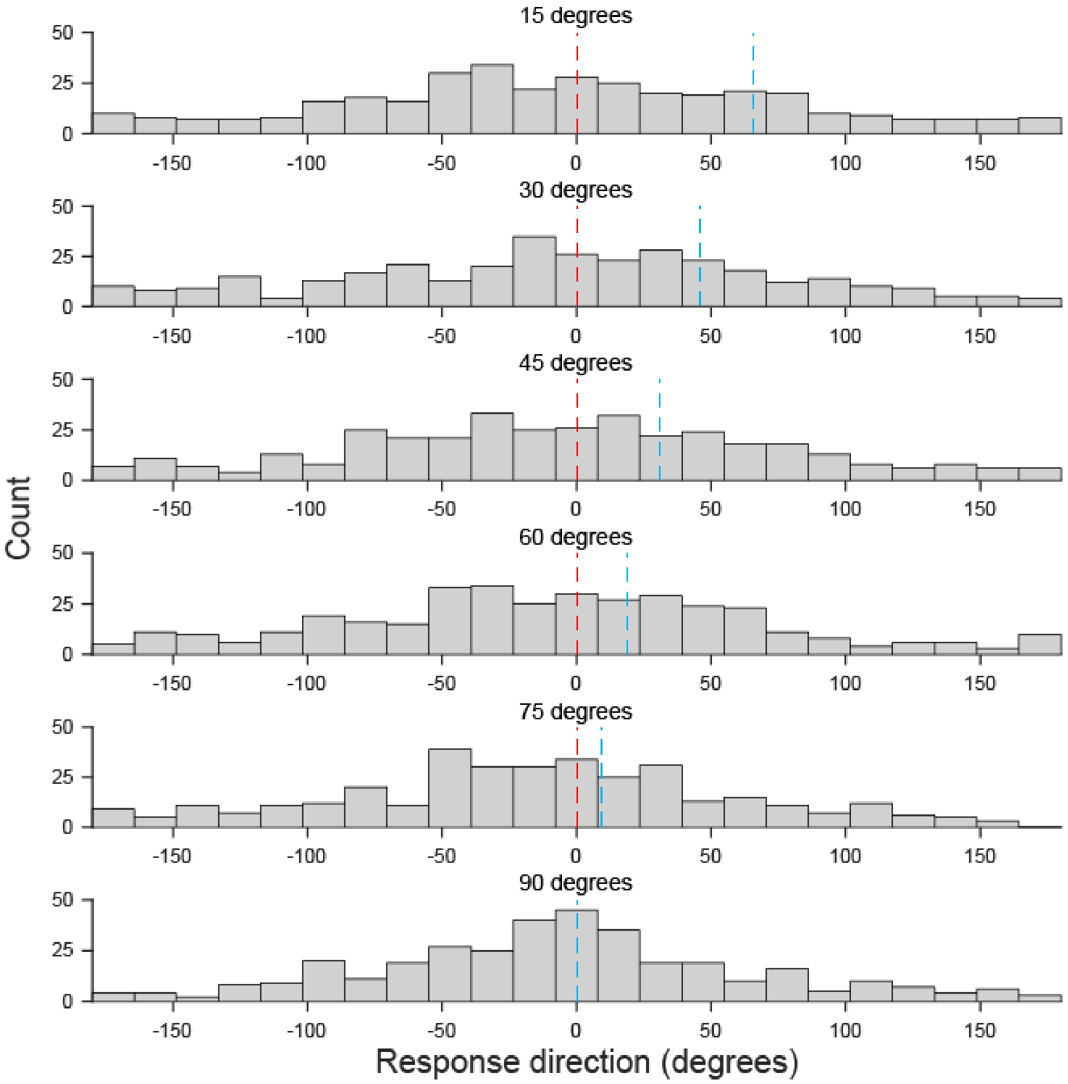

Supplementary Figure S4. The pigeons' responses were not deviated from the VA direction as difference of direction between components increased. Histograms represents distributions of response direction relative to the VA direction of plaid for each condition about difference of directions between components. Red and blue dotted lines represent the VA and IOC direction of plaid, respectively.
